# Socioeconomic Deprivation and Kidney Transplant Outcomes

**DOI:** 10.1016/j.ekir.2025.10.024

**Published:** 2025-11-05

**Authors:** Lukas Ponette, Karolien Wellekens, Priyanka Koshy, Arthur Vranken, Thomas Vanhoutte, Dirk Kuypers, Maarten Naesens, Maarten Coemans

**Affiliations:** 1 Department of Microbiology, Immunology and Transplantation, Nephrology and Renal Transplantation Research Group, KU Leuven, Leuven, Belgium; 2 Department of Nephrology and Renal Transplantation, University Hospitals Leuven, Leuven, Belgium; 3 Department of Pathology, University Hospitals Leuven, Leuven, Belgium; 4 Department of Public Health and Primary Care, Leuven Biostatistics and Statistical Bioinformatics Centre, KU Leuven, Leuven, Belgium

**Keywords:** graft failure, kidney transplantation, mortality, rejection, socioeconomic deprivation

## Abstract

**Introduction:**

Socioeconomic deprivation adversely affects health outcomes, including those after kidney transplantation. Retrospective studies, including 92,844 patients in the US and 19,103 and 621 in 2 UK cohorts, reported higher mortality and graft rejection among deprived individuals. The persistence of these disparities in the UK, despite having universal health care, suggests that insurance coverage alone does not eliminate inequities. Whether similar patterns exist in Belgium remains unclear.

**Methods:**

We studied 1891 kidney transplant recipients (2004–2021) at University Hospitals Leuven, by assessing socioeconomic deprivation using the Belgian Index of Multiple Deprivation (BIMD). The outcomes were all-cause graft failure, graft failure, mortality, and rejection, analyzed with Cox and competing risks models.

**Results:**

Socioeconomic deprivation was associated with all-cause graft failure in univariable analysis (hazard ratio [HR]: 1.07, 95% confidence interval [CI]: 1.00–1.14, *P* = 0.043), but not after adjustment (adjusted HR [aHR]: 1.03, 95% CI: 0.97–1.10, *P* = 0.315). No significant associations were observed for graft failure (aHR: 1.04, 95% CI: 0.94–1.15, *P* = 0.467) or mortality (aHR: 1.04, 95% CI: 0.96–1.13, *P* = 0.366). In contrast, rejection risk differed significantly across deprivation groups (*P* = 0.030), driven by higher rates of T-cell–mediated rejection (TCMR) (aHR: 1.08, 95% CI: 1.00–1.16, *P* = 0.036), whereas antibody-mediated rejection (AMR) showed no association (aHR: 1.00, 95% CI: 0.88–1.15, *P* = 0.984).

**Conclusion:**

Socioeconomic deprivation in Belgium was associated with rejection, specifically TCMR, and univariably with all-cause graft failure. We lacked evidence of associations with graft failure or mortality separately. These findings suggest that universal health care may mitigate some adverse posttransplantation outcomes because of socioeconomic deprivation. Future studies should evaluate whether deprivation affects access to transplantation itself.

## Graphical abstract

**Figure:**
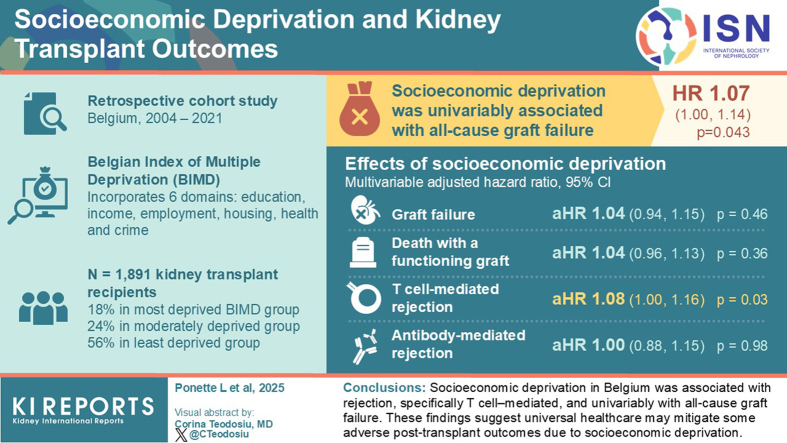

Multiple studies have demonstrated that life expectancy varies significantly with socioeconomic status, even in high-income countries.^1^^,^^2^ This disparity is attributed to unequal access to health care, differences in living conditions, education, employment opportunities, and varying levels of social support.^3^^,^^4^ In the US kidney transplant population, higher socioeconomic status correlated with improved graft and patient survival.^5^^,^^6^ These studies identified a link between insurance type and graft failure, suggesting that limited access to care or differences in public versus private insurance coverage contribute to these outcomes. Similar disparities were observed in the UK, which has a universal health care system, including differences in patient survival^7^ and acute rejection^8^ rates between socioeconomic groups, indicating that such disparities can persist despite universal coverage. Whether similar patterns occur in Belgium’s universal health care system remains unknown. Indeed, in contrast to the US, where 29% to 41% of transplant recipients report financial strain because of the high cost of immunosuppressive medication,^9^^,^^10^ health insurance in Belgium is mandatory and financed by social contributions, proportional to income.^11^ Although Belgium’s overall out-of-pocket health spending is higher than that of its neighboring countries, it is close to the European Union’s average, with higher incomes bearing most of its increase over the years.^12^

Socioeconomic deprivation is a multidimensional concept. Goldfarb-Rumyantzev *et al.*^6^ measured socioeconomic deprivation using 3 variables, namely primary source of payment for medical services, education level, and citizenship or immigration status. In Belgium, the BIMD was developed to provide a more comprehensive assessment.^13^ The BIMD is a standardized, neighborhood-level evaluation of deprivation based on income, employment, education, housing, health, and crime.

For this study, we linked the BIMD scores to the largest kidney transplant cohort in Belgium and assessed the impact of socioeconomic deprivation on posttransplant outcomes. We hypothesized that greater socioeconomic deprivation, as indicated by higher BIMD scores, are associated with higher rates of all-cause graft failure, graft failure, mortality and rejection.

## Methods

### Study Population and Data Collection

This cohort study included all single adult kidney transplants performed at University Hospitals Leuven between March 2004 and May 2021. For all recipients, clinical data were prospectively collected during routine follow-up until June 11, 2022. Patients with additional immunological risk (e.g., prior transplantation), of other than White European ethnicity, or aged < 35 years received additional induction therapy with interleukin-2 receptor blockers. Biopsy data were collected per protocol at 3, 12, and 24 months posttransplantation and when clinically indicated. Patients transplanted before October 2005, November 2008, and January 2010 were additionally invited for protocol biopsies at 48, 36, and 60 months, respectively, as part of the center’s historical long-term follow-up protocol. Biopsies were evaluated using semiquantitative Banff lesion scores, following the Reference Guide to the Banff 2022 Classification (https://banfffoundation.org/central-repository-for-banff-classification-resources-3/)) and were subsequently classified according to the Banff 2022 criteria^14^ into 3 diagnostic categories as follows: AMR (including acute, chronic active, and chronic AMR), TCMR (including acute and chronic active TCMR), and no rejection. Borderline TCMR or other AMR/microvascular inflammation phenotypes (probable AMR; microvascular inflammation, donor-specific antibody [DSA]–negative and C4d-negative) were not considered in the definition of rejection as end point. The study was approved by the University Hospitals Leuven Ethics Committee (S64006, NCT06505200) and conducted in accordance with the Declaration of Helsinki.

### Outcomes

The following 4 end points were assessed: (i) all-cause graft failure, defined as overall loss of graft function or patient death; (ii) graft failure, defined as the need for dialysis or retransplantation;(iii) mortality, defined as death with a functioning graft; (iv) and rejection, defined as AMR and/or TCMR. All time-to-event analyses started on the day of transplantation. All-cause graft failure, graft failure and recipient death were censored at last follow-up, at 10 years when follow-up was longer or events occurred later, or on June 11, 2022 (administrative censoring date). Rejection data were censored at the last biopsy before 5 years posttransplant.

### BIMD

The BIMD, developed by Otavova *et al.*^13^ provides a relative measure of socioeconomic deprivation at the level of a statistical sector, which is the smallest administrative unit for subdividing municipalities in Belgium.^15^ The BIMD score ranges from 0 (least deprived) to 100 (most deprived) and incorporates 6 weighted domains: education (25%), income (20%), employment (20%), housing (15%), health (15%), and crime (5%) (Supplementary Table S1). Next to the overall score, the BIMD provides a rank for each statistical sector (*n* = 18,764) and a corresponding decile (1 = most deprived; 10 = least deprived). For this study, both the continuous BIMD scores and a categorization based on deciles were used. According to the BIMD deciles, patients were classified into 3 groups as follows: (i) “most deprived” (1–3), (ii) “moderately deprived” (4–6), and (iii) “least deprived” (7–10).

### Statistical Analysis

This study followed the STROBE guidelines (Supplementary Table S2). Inclusion required complete data on donor or recipient age and biological sex, repeat transplantation, ethnicity (White European vs. other), recipient pretransplant diabetes, donor type (brain death, circulatory death, and living), cold ischemia time, presence or absence of anti–human leukocyte antigen DSA (HLA-DSA), number of HLA-ABDR mismatches, transplant year, baseline immunosuppression, and BIMD score. Patients without adequate posttransplant biopsies were excluded from the rejection analyses. Continuous variables were reported as means and SDs or in medians and interquartile ranges (IQRs). Categorical variables were summarized as frequencies and proportions. Correlations between BIMD scores and its domains were assessed using the Pearson correlation. BIMD group comparisons were performed using 1-way analysis of variance, Kruskal-Wallis, or chi-square tests, as appropriate. The cumulative incidences of graft failure and death with functioning graft were estimated across the 3 BIMD groups using the Aalen-Johansen method, treating both outcomes as competing events,^16^ and using Gray’s test for group comparisons. All-cause graft failure and time-to-first-rejection was analyzed using the Kaplan-Meier estimator, with BIMD group comparisons conducted via the log-rank test. Univariable and multivariable (cause-specific) Cox proportional hazards models assessed the association of the continuous BIMD score with the rate of all-cause graft failure, graft failure, death with functioning graft, and rejection. In the univariable models, we visually assessed proportionality and linearity with a plot of the weighted Schoenfeld residuals and Martingale residuals, respectively, each time including a B-spline function (degree = 3, knots = 5) to evaluate deviations from a horizontal line. Multivariable adjustment was done for recipient age, recipient biological sex, recipient ethnicity, recipient pretransplant diabetes, repeat transplantation, transplant year, donor age, donor biological sex, donor type (donation after brain death, donation after circulatory death, living), pretransplant HLA-DSA presence or absence, the number of HLA-ABDR mismatches and baseline immunosuppression. Univariable and multivariable Fine and Gray competing risks models were fitted to assess the association of the continuous BIMD scores with the cumulative incidence of graft failure and death with functioning graft. In addition, rejection analyses were conducted separately for TCMR and AMR subtypes. All Cox as well as Fine and Gray models were performed for each BIMD domain (income, employment, education, housing, health, and crime), treating these as continuous scores (0–100). The statistical analyses were conducted in R (R version 4.4.1, http://www.r-project.org), using 2-sided hypothesis tests and a significance threshold of *P* < 0.05. The proportionality and linearity graphs were created with SAS 9.4 (SAS Institute, Cary, NC).

## Results

### Study Cohort Characteristics

A total of 1891 transplants were eligible for analysis. Of these, 21 were excluded because of unavailable BIMD scores, leaving 1870 transplants available for the graft failure and mortality analyses (Supplementary Figure S1). For the rejection analysis, 93 additional exclusions were made because of no or inadequate posttransplant biopsies, resulting in a sample of 1777 transplants. For these, 5795 biopsies were included, of which 4296 were protocol and 1499 indication biopsies. The median follow-up time was 6.20 (IQR: 3.06–9.99) years for the graft failure and mortality cohort, and 1.13 (IQR: 0.25–2.05) years for the rejection cohort (Table 1).

**Table 1:** Demographics of the graft failure/mortality (*n* = 1870) and rejection cohort (*n* = 1777)

Characteristics	Total (*n* = 1870)	Most deprived (*n* = 347)	Moderately deprived (*n* = 463)	Least deprived (*n* = 1060)	*P*-value
Recipient					
Age (yrs), mean ± SD	54.6 ± 12.8	53.9 ± 12.5	54.9 ± 12.9	54.7 ± 12.9	0.549
Female, *n* (%)	687 (36.7%)	133 (38.3%)	169 (36.5%)	385 (36.3%)	0.791
Repeat transplantation, *n* (%)	280 (15.0%)	55 (15.9%)	75 (16.2%)	150 (14.2%)	0.517
White European ethnicity, *n* (%)	1801 (96.3%)	312 (89.9%)	447 (96.5%)	1042 (98.3%)	< 0.001
Pretransplant diabetes, *n* (%)	337 (18.0%)	95 (27.4%)	80 (17.3%)	162 (15.3%)	< 0.001
Donor					
Age (yrs), mean ± SD	49.2 ± 14.3	48.5 ± 14.5	49.6 ± 14.3	49.3 ± 14.3	0.537
Female, *n* (%)	849 (45.4%)	161 (46.4%)	206 (44.5%)	482 (45.5%)	0.863
Living donation, *n* (%)	142 (7.6%)	30 (8.6%)	35 (7.6%)	77 (7.3%)	0.902
Donation after circulatory death, *n* (%)	369 (19.7%)	70 (20.2%)	94 (20.3%)	205 (19.3%)	
Donation after brain death, *n* (%)	1359 (72.7%)	247 (71.2%)	334 (72.1%)	778 (73.4%)	
Transplant					
Cold ischemia time (h), median (IQR)^a^	13.26 (9.55–16.70)	13.45 (9.23–16.98)	13.63 (10.00–16.83)	13.07 (9.39–16.37)	0.577
Pretransplant HLA-DSA, *n* (%)	150 (8.0%)	35 (10.1%)	40 (8.6%)	75 (7.1%)	0.171
De novo HLA-DSA, *n* (%)^b^	101 (5.5%)	23 (6.8%)	28 (6.1%)	50 (4.8%)	0.325
No. of HLA-ABDR mismatches (0-6)	2.6 ± 1.3	2.7 ± 1.1	2.6 ± 1.3	2.5 ± 1.3	0.180
TAC-MMF-CS, *n* (%)	1714 (91.7%)	328 (94.5%)	420 (90.7%)	966 (91.1%)	0.098
Induction therapy, *n* (%)	827 (44.2%)	199 (57.3%)	220 (47.5%)	408 (38.5%)	< 0.001
Transplant year, median (IQR)	2012 (2008–2016)	2012 (2008–2016)	2012 (2008–2017)	2013 (2008–2016)	0.123
Posttransplant					
Follow-up time (yrs), median (IQR)	6.20 (3.06–9.99)	5.76 (3.00–9.24)	6.21 (3.25–10.70)	6.30 (3.03–10.16)	0.225
All-cause graft failure, *n* (%)	610 (32.6%)	128 (36.9%)	152 (32.8%)	330 (31.1%)	
Graft failure, *n* (%)	252 (13.5%)	53 (15.3%)	66 (14.3%)	133 (12.5%)	
Death with functioning graft, *n* (%)	358 (19.1%)	75 (21.6%)	86 (18.6%)	197 (18.6%)	
BIMD domains					
Overall, median (IQR)	13.68 (9.18-22.40)	35.79 (26.43-72.69)	19.66 (15.65-26.38)	9.82 (1.47-15.63)	< 0.001
Income, median (IQR)	12.85 (6.23–25.81)	39.65 (3.35–98.82)	18.51 (0.02–97.86)	7.52 (0.22–62.42)	< 0.001
Employment, median (IQR)	13.43 (5.74–24.63)	36.91 (2.44–90.73)	21.01 (0.48–52.70)	7.35 (0.01–32.96)	< 0.001
Education, median (IQR)	12.47 (4.56–28.24)	52.54 (18.06–99.45)	22.65 (0.86–50.38)	5.95 (0.01–29.07)	< 0.001
Housing, median (IQR)	12.89 (7.41–22.79)	20.67 (1.61–95.36)	16.18 (1.09–75.94)	11.03 (0.02–66.92)	< 0.001
Crime, median (IQR)	18.01 (7.41–22.79)	45.17 (5.75–100.00)	24.61 (0.43–88.51)	10.58 (0.08–97.20)	< 0.001
Health, median (IQR)	7.41 (7.41–22.79)	7.00 (0.17–66.51)	8.48 (0.09–73.28)	6.97 (0.02–59.09)	< 0.001
Rejection cohort	(*n* = 1777)	(*n* = 326)	(*n* = 446)	(*n* = 1005)	
Follow-up time (yrs), median (IQR)	1.13 (0.25–2.05)	1.07 (0.25–2.05)	1.11 (0.24–2.04)	1.42 (0.26–2.05)	0.285
Overall rejection, *n* (%)	518 (29.2%)	111 (34.0%)	134 (30.0%)	273 (27.2%)	
AMR, *n* (%)	160 (9.0%)	30 (9.2%)	44 (9.9%)	86 (8.6%)	
TCMR, *n* (%)	460 (25.9%)	99 (30.4%)	123 (27.6%)	238 (23.7%)	

AMR, antibody-mediated rejection; BIMD, Belgian index of multiple deprivation; CS, corticosteroids; DSA, antigen donor-specific antibody. HLA-DSA, anti–human leukocyte antigen DSA; IQR, interquartile range; MMF, mycophenolate mofetil; TAC, tacrolimus; TCMR, T cell mediated rejection.

a 2 missing values.

b 43 patients had no DSA follow-up and were excluded from these numbers.

**Figure 1:**
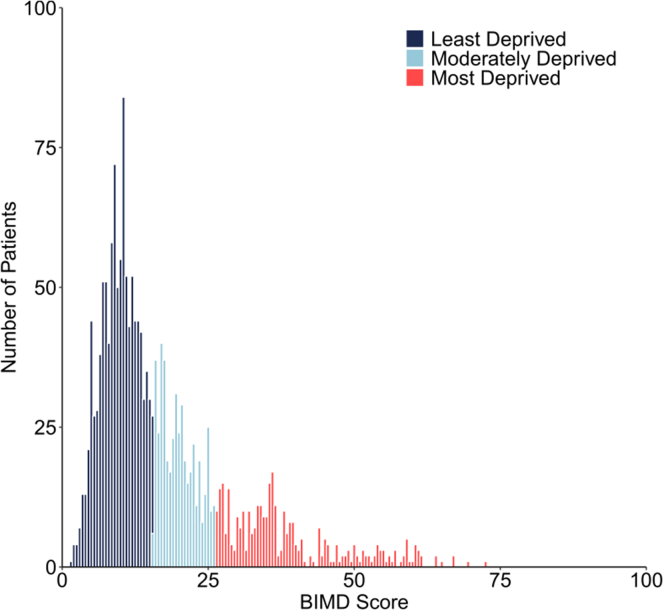
Distribution of BIMD score by deprivation groups. Recipients were classified into 3 groups based on BIMD deciles: “Most deprived” (deciles 1–3), “Moderately deprived” (4–6), and “Least deprived” (7–10). A BIMD score of 100 corresponds to the most deprived, whereas 0 corresponds to the least deprived. BMID, Belgian Index of Multiple Deprivation.

Of the 1870 transplant recipients, 347 (18.6%) were included in the most deprived group, 463 (24.8%) were moderately deprived, and 1060 (56.7%) recipients belonged to the least deprived group, which demonstrates the skewed distribution of the BIMD scores (Table 1 and Figure 1). The most deprived group had the highest proportion of non–White European recipients (10.1% vs. 1.7% in the least deprived, overall *P* < 0.001), the greatest prevalence of diabetes (27.4% vs. 15.3%, overall *P* < 0.001), and the highest likelihood of receiving induction therapy (57.3% vs. 38.5%, overall *P* < 0.001).

Naturally, the 3 deprivation groups differed significantly in all BIMD domains (income, employment, education, housing, health, and crime) (Table 1 and Supplementary Figure S2); however, on the continuous scale, BIMD scores showed varying correlations with these domains. The BIMD score was highly correlated with education (*R* = 0.94, *P* < 0.001), employment (*R* = 0.81, *P* < 0.001), and income (*R* = 0.82, *P* < 0.001); moderately correlated with crime (*R* = 0.58, *P* < 0.001) and housing (*R* = 0.37, *P* < 0.001), and had low correlation with health (*R* = 0.12, *P* < 0.001) (Supplementary Figure S3).

### Effect of Socioeconomic Deprivation on All-Cause Graft Failure, Graft Failure, and Mortality

**Figure 2:**
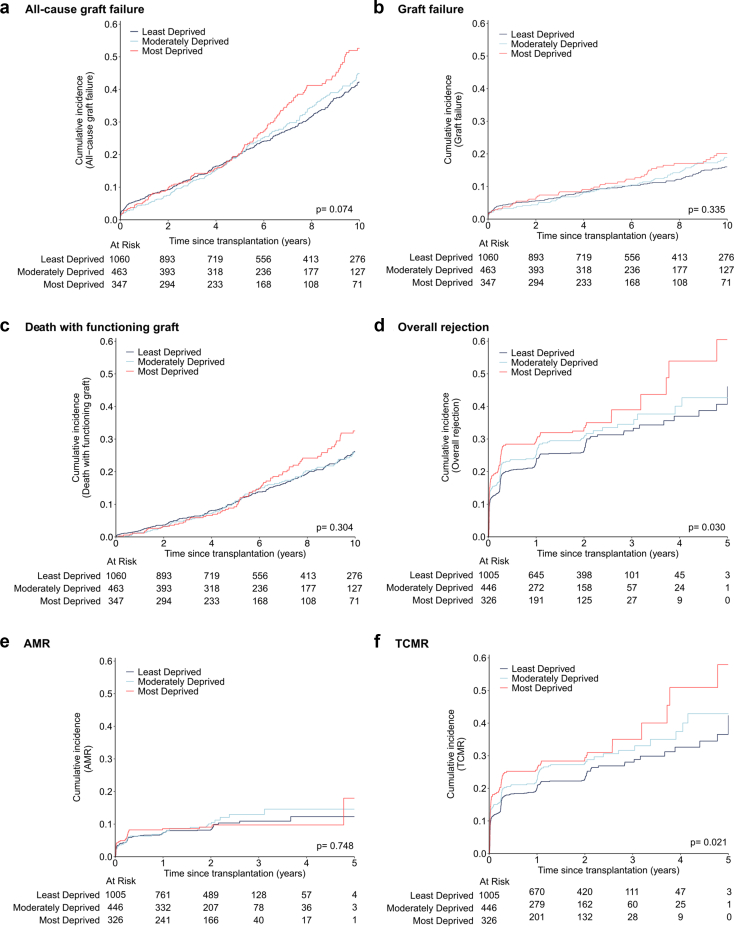
Cumulative incidence of (a) all-cause graft failure, (b) graft failure, (c) death with functioning graft, (d) overall rejection, (e) AMR, and (f) TCMR, by groups of socioeconomic deprivation. Pairwise comparisons, least vs. most deprived: (a) *P* = 0.023, (b) *P* = 0.159, (c) *P* = 0.144, (d) *P* = 0.009, (e) *P* = 0.705, (f) *P* = 0.009; least vs. moderately deprived: (a) *P* = 0.624, (b) *P* = 0.425, (c) *P* = 0.937, (d) *P* = 0.195, (e) *P* = 0.459, (f) *P* = 0.089; moderately vs. most deprived: (a) *P* = 0.107, (b) *P* = 0.531, (c) *P* = 0.188, (d) *P* = 0.207, (e) *P* = 0.824, (f) *P* = 0.328. AMR, antibody-mediated rejection; TCMR, T cell mediated rejection.

In total, 610 recipients experienced all-cause graft failure; 252 (13.5%) developed graft failure and 358 (19.1%) died with a functioning graft (Table 1). Ten-year cumulative incidences of all-cause graft failure were 42.2% (95% CI: 38.5%–45.9%) in the least deprived group, 44.9% (95% CI: 39.2%–50.4%) in the moderately deprived group, and 52.6% (45.5%–59.2%) in the most deprived group (*P* = 0.074). When outcomes were analyzed separately, the cumulative incidence of graft failure did not differ significantly between deprivation groups (*P* = 0.335), with 10-year estimates of 16.0% (95% CI: 13.5%–18.7%) for the least deprived, 18.9% (14.9%–23.4%) for the moderately deprived, and 20.1% (15.3%–25.4%) for the most deprived (Figure 2). Similarly, death with a functioning graft showed no significant differences (*P* = 0.304), with 10-year estimates of 26.2% (23.0%–29.5%), 25.9% (21.2%–30.9%), and 32.5% (26.3%–38.9%), respectively.

Using the continuous BIMD score, all-cause graft failure was associated in univariable analysis (HR: 1.07 per 10-point increase, 95% CI: 1.00–1.14, *P* = 0.043); however, this effect was not sustained after multivariable adjustment (HR: 1.03, 95% CI: 0.97–1.10, *P* = 0.315) (Supplementary Table S3). For graft failure and death with a functioning graft, both Cox and Fine and Gray competing risks models showed no significant associations (Cox univariable HRs: 1.07 [95% CI: 0.97–1.17], *P* = 0.199; and 1.07 [95% CI 0.98–1.16], *P* = 0.117; Fine and Gray univariable subdistribution HRs 1.06 [95% CI: 0.96–1.16], *P* = 0.230; and 1.06 [95% CI: 0.98–1.15], *P* = 0.165) (Table 2). No severe deviations from linearity or proportional hazards assumptions were detected (Supplementary Figures S4 and S5).

**Table 2:** Association of BIMD score with graft failure and mortality (*n* = 1870)

Graft failure	Cox model				Fine and Gray			
	Univariable HR (95%CI)	*P*-value	Multivariable HR (95%CI)	*P*-value	Univariable sHR (95%CI)	*P*-value	Multivariable sHR (95%CI)	*P*-value
BIMD score, per 10	1.07 (0.97–1.17)	0.199	1.04 (0.94–1.15)	0.467	1.06 (0.96–1.16)	0.230	1.03 (0.94–1.14)	0.484
Recipient age, per 10 yrs	1.02 (0.93–1.13)	0.648	0.89 (0.80–0.99)	0.039	0.96 (0.87–1.07)	0.463	0.84 (0.76–0.94)	0.002
Recipient female	0.79 (0.62–1.02)	0.069	0.85 (0.66–1.10)	0.224	0.78 (0.61–1.00)	0.048	0.86 (0.67–1.10)	0.225
Other ethnicity (ref = White European)	1.23 (0.55–2.77)	0.618	1.35 (0.58–3.10)	0.486	1.26 (0.57–2.79)	0.566	1.43 (0.61–3.35)	0.407
Recipient pretransplant diabetes	1.47 (1.09–1.99)	0.012	1.50 (1.09–2.04)	0.012	1.29 (0.96–1.73)	0.097	1.37 (1.01–1.86)	0.041
Repeat transplantation	1.70 (1.26–2.30)	< 0.001	1.52 (1.08 –2.13)	0.015	1.65 (1.23–2.21)	< 0.001	1.41 (1.01–1.98)	0.044
Transplant year	0.98 (0.95–1.01)	0.176	0.98 (0.95–1.01)	0.230	0.97 (0.94–1.00)	0.025	0.97 (0.94–1.00)	0.071
Donor age, per 10 yrs	1.21 (1.10–1.33)	< 0.001	1.24 (1.12–1.38)	< 0.001	1.18 (1.06–1.30)	0.002	1.24 (1.11–1.38)	< 0.001
Donor female	0.95 (0.74–1.22)	0.704	1.08 (0.83–1.39)	0.577	0.95 (0.75–1.22)	0.691	1.06 (0.82–1.36)	0.649
Living donation (ref = DBD)	0.48 (0.26–0.91)	0.024	0.40 (0.21–0.78)	0.007	0.52 (0.28–0.97)	0.039	0.41 (0.22–0.77)	0.006
Donor DCD (ref = DBD)	0.76 (0.54–1.07)	0.120	0.80 (0.57–1.14)	0.227	0.77 (0.55–1.08)	0.124	0.82 (0.59–1.16)	0.260
Pretransplant HLA-DSA	2.77 (2.01–3.81)	< 0.001	2.22 (1.56–3.16)	< 0.001	2.71 (2.00–3.67)	< 0.001	2.21 (1.56–3.14)	< 0.001
Number of HLA-ABDR mm	1.15 (1.04–1.28)	0.005	1.15 (1.03–1.28)	0.011	1.13 (1.01–1.26)	0.026	1.14 (1.01–1.27)	0.032
TAC-MMF-CS (ref = other)	1.15 (0.74–1.79)	0.524	1.07 (0.68–1.69)	0.770	1.09 (0.70–1.68)	0.710	1.03 (0.64–1.64)	0.913
Mortality								
BIMD score, per 10	1.07 (0.98–1.16)	0.117	1.04 (0.96–1.13)	0.366	1.06 (0.98–1.15)	0.165	1.03 (0.95–1.11)	0.467
Recipient age per 10 yrs	2.28 (2.03–2.56)	< 0.001	2.21 (1.95–2.52)	< 0.001	2.16 (1.93–2.42)	< 0.001	2.15 (1.89–2.45)	< 0.001
Recipient female	1.16 (0.93–1.44)	0.184	1.04 (0.83–1.30)	0.752	1.19 (0.96–1.47)	0.111	1.03 (0.84–1.27)	0.765
Other ethnicity (ref = White European)	0.91 (0.48–1.71)	0.765	0.85 (0.44–1.64)	0.627	0.92 (0.50–1.69)	0.785	0.82 (0.46–1.48)	0.517
Recipient pretransplant diabetes	2.75 (2.20–3.43)	< 0.001	1.99 (1.58–2.51)	< 0.001	2.52 (2.04–3.11)	< 0.001	1.83 (1.46–2.29)	< 0.001
Repeat transplantation	1.23 (0.93–1.63)	0.155	1.82 (1.34–2.48)	0.001	1.12 (0.85–1.48)	0.409	1.56 (1.18–2.04)	0.002
Transplant year	1.05 (1.02–1.08)	0.001	1.03 (1.00–1.06)	0.061	1.03 (1.01–1.06)	0.013	1.01 (0.99–1.04)	0.349
Donor age, per 10 yrs	1.22 (1.13–1.32)	< 0.001	0.98 (0.91–1.06)	0.672	1.18 (1.08–1.28)	< 0.001	0.94 (0.88–1.02)	0.129
Donor female	0.93 (0.76–1.15)	0.494	1.00 (0.81–1.25)	0.971	0.94 (0.76–1.15)	0.517	0.97 (0.79–1.19)	0.752
Living donation (ref = DBD)	0.27 (0.14–0.55)	< 0.001	0.60 (0.29–1.23)	0.162	0.29 (0.15–0.60)	< 0.001	0.67 (0.32–1.40)	0.290
Donor DCD (ref = DBD)	0.82 (0.62–1.08)	0.156	0.89 (0.67–1.19)	0.445	0.84 (0.63–1.11)	0.211	0.96 (0.73–1.26)	0.753
Pretransplant HLA-DSA	1.13 (0.77–1.65)	0.540	0.97 (0.65–1.45)	0.872	0.94 (0.65–1.37)	0.762	0.82 (0.57–1.16)	0.263
No. of HLA-ABDR mm	1.16 (1.06–1.26)	< 0.001	1.07 (0.98–1.17)	0.125	1.13 (1.03–1.22)	0.007	1.03 (0.95–1.12)	0.435
TAC-MMF-CS (ref = other)	1.46 (0.99–2.17)	0.058	1.20 (0.79–1.81)	0.394	1.41 (0.96–2.07)	0.078	1.27 (0.88–1.84)	0.207

AMR, antibody-mediated rejection; BIMD, Belgian index of multiple deprivation; CS, corticosteroids; DBD, donation after brain death; DCD, donation after circulatory death; HLA-DSA, anti–human leukocyte antigen donor-specific antibodies; HR, hazard ratio; IQR, interquartile range; MMF, mycophenolate mofetil; sHR, subdistribution HR ; TAC, tacrolimus.

Hazard and subdistribution hazard ratios for graft failure and mortality, up to 10 years posttransplantation, including the BIMD score as a continuous variable (0 = least deprived; 100 = most deprived).

Apart from the lack of a significant association between BIMD and graft failure or mortality, several well-established clinical factors significantly influenced transplant outcomes (Table 2). All-cause graft failure was associated in both univariable and multivariable analyses with recipient age, recipient pretransplant diabetes, repeat transplantation, donor age, living donation, pretransplant HLA-DSA, and HLA-ABDR mismatches. Graft failure was consistently associated with recipient pretransplant diabetes, repeat transplantation, donor age, living donation, pretransplant HLA-DSA, and HLA-ABDR mismatches in both univariable and multivariable Cox models; recipient age was significant only in the multivariable analysis. Mortality was consistently associated with recipient age and recipient pretransplant diabetes; whereas donor age, living donation, HLA-ABDR mismatches, and transplant year were significant only in univariable models, and repeat transplantation only in the multivariable model.

### Effect of Socioeconomic Deprivation on Rejection

A total of 518 recipients (29.2%) experienced rejection at least once during the 5-year follow-up period. The cumulative incidence of rejection within 5 years posttransplant was significantly different between the deprivation groups (*P* = 0.030) (Figure 2). Indeed, the 5-year cumulative incidence estimates were 46% (33.3%–57.9%) in the least deprived group, 42.7% (33.3%–51.7%) in the moderately deprived group, and 60.5% (38.9%–76.5%) in the most deprived group. Accordingly, in the univariable Cox model, each 10-point increase in the BIMD score was associated with a 10.2% increase of the rejection rate (HR: 1.10, 95% CI: 1.03–1.18, *P* = 0.004) (Table 3). Moreover, no notable deviation from linearity and proportionality was detected (Supplementary Figures S4 and S5). In the multivariable analysis, adjusted for recipient age, recipient biological sex, recipient ethnicity, recipient pretransplant diabetes, repeat transplantation, transplant year, donor age, donor biological sex, donor type (donation after brain death, donation after circulatory death, living), pretransplant HLA-DSA, HLA-ABDR mismatches, baseline immunosuppression, and BIMD scores remained significantly associated with rejection (HR: 1.07, 95% CI: 1.00–1.15, *P* = 0.040). The association between BIMD and overall rejection was explained by an increased rate of TCMR with increasing BIMD (univariable HR: 1.10, 95% CI: 1.02–1.18, *P* = 0.010). No significant association was observed with AMR (univariable HR: 1.07, 95% CI: 0.95–1.21, *P* = 0.288). No severe deviations from linearity or proportionality were observed (Supplementary Figures S4 and S5).

**Table 3:** Association of BIMD score with overall rejection, TCMR and AMR (*n* = 1777)

Overall rejection	Cox model			
	Univariable HR (95% CI)	*P*-value	Multivariable HR (95% CI)	*P*-value
BIMD score, per 10	1.10 (1.03–1.18)	0.004	1.07 (1.00–1.15)	0.040
Recipient age, per 10 yrs	1.00 (0.93–1.07)	0.993	0.92 (0.85–0.99)	0.023
Recipient female	0.81 (0.68–0.97)	0.021	0.89 (0.74–1.06)	0.189
Other ethnicity (ref = White European)	1.38 (0.81–2.35)	0.235	1.64 (0.95–2.84)	0.078
Recipient pretransplant diabetes	1.35 (1.10–1.67)	0.005	1.35 (1.09–1.69)	0.007
Repeat transplantation	1.33 (1.06–1.67)	0.014	1.11 (0.86–1.42)	0.421
Transplant year	0.96 (0.94–0.97)	< 0.001	0.96 (0.94–0.98)	< 0.001
Donor age, per 10 yrs	1.05 (0.99–1.12)	0.129	1.08 (1.01–1.15)	0.029
Donor female	0.87 (0.74–1.04)	0.126	0.91 (0.76–1.09)	0.300
Living donation (ref = DBD)	0.74 (0.52–1.06)	0.098	0.63 (0.43–0.93)	0.018
Donor DCD (ref = DBD)	0.85 (0.68–1.07)	0.160	0.97 (0.77–1.23)	0.825
Pretransplant HLA-DSA	4.15 (3.33–5.17)	< 0.001	3.75 (2.96–4.76)	< 0.001
Number of HLA-ABDR mismatches	1.15 (1.08–1.23)	< 0.001	1.17 (1.08–1.26)	< 0.001
TAC-MMF-CS (ref = other)	0.76 (0.58–1.01)	0.056	0.80 (0.60–1.07)	0.130
TCMR				
BIMD score, per 10	1.10 (1.02–1.18)	0.010	1.08 (1.00–1.16)	0.036
Recipient age, per 10 yrs	1.01 (0.94–1.08)	0.874	0.91 (0.84–0.99)	0.030
Recipient female	0.87 (0.72–1.05)	0.146	0.89 (0.73–1.08)	0.222
Other ethnicity (ref = White European)	2.20 (1.10–4.43)	0.027	2.56 (1.26–5.24)	0.010
Recipient pretransplant diabetes	1.45 (1.17–1.81)	0.001	1.47 (1.17–1.85)	0.001
Repeat transplantation	0.98 (0.75–1.27)	0.860	0.93 (0.70–1.23)	0.620
Transplant yr	0.96 (0.94–0.98)	< 0.001	0.97 (0.95–0.99)	0.002
Donor age, per 10 yrs	1.04 (0.98–1.11)	0.226	1.05 (0.98–1.13)	0.184
Donor female	0.88 (0.73–1.05)	0.155	0.90 (0.74–1.08)	0.259
Living donation (ref = DBD)	0.68 (0.46–1.00)	0.052	0.58 (0.38–0.87)	0.009
Donor DCD (ref = DBD)	0.85 (0.67–1.08)	0.183	0.94 (0.74–1.21)	0.634
Pretransplant HLA-DSA	2.05 (1.56–2.70)	< 0.001	1.94 (1.45–2.60)	<0.001
No. of HLA-ABDR mismatches	1.16 (1.08–1.25)	< 0.001	1.20 (1.10–1.30)	<0.001
TAC-MMF-CS (ref = other)	0.71 (0.54–0.95)	0.021	0.74 (0.55–1.00)	0.048
AMR				
BIMD score, per 10	1.07 (0.95–1.21)	0.288	1.00 (0.88–1.15)	0.984
Recipient age, per 10 yrs	0.86 (0.76–0.96)	0.009	0.84 (0.74–0.96)	0.008
Recipient female	0.69 (0.51–0.94)	0.020	1.02 (0.74–1.42)	0.884
Other ethnicity (ref = White European)	0.69 (0.34–1.41)	0.306	0.83 (0.39–1.81)	0.646
Recipient pretransplant diabetes	1.13 (0.76–1.68)	0.550	1.19 (0.79–1.81)	0.407
Repeat transplantation	3.73 (2.70–5.15)	< 0.001	1.55 (1.04–2.32)	0.030
Transplant yr	0.93 (0.89–0.96)	< 0.001	0.94 (0.90–0.97)	0.001
Donor age, per 10 yrs	1.00 (0.90–1.12)	0.964	1.13 (1.00–1.27)	0.042
Donor female	0.84 (0.62–1.15)	0.276	0.92 (0.67–1.28)	0.632
Living donation (ref = DBD)	1.33 (0.78–2.20)	0.277	1.20 (0.61–2.37)	0.605
Donor DCD (ref = DBD)	0.66 (0.42–1.04)	0.075	1.10 (0.68–1.76)	0.697
Pretransplant HLA-DSA	23.97 (17.42–32.98)	< 0.001	20.10 (14.00–28.85)	< 0.001
No. of HLA-ABDR mismatches	1.10 (0.97–1.24)	0.141	1.07 (0.93–1.23)	0.355
TAC-MMF-CS (ref = other)	0.93 (0.55–1.59)	0.804	0.86 (0.49–1.51)	0.593

AMR, antibody-mediated rejection; BIMD, Belgian index of multiple deprivation; CS, corticosteroids; DBD, donation after brain death; DCD, donation after circulatory death; HLA-DSA, anti–human leukocyte antigen donor-specific antibodies; IQR, interquartile range; MMF, mycophenolate mofetil; TAC, tacrolimus; TCMR, T-cell–mediated rejection.

Hazard ratios for overall rejection, TCMR, and AMR, up to 5 years posttransplantation, including the BIMD score as a continuous variable (0 = least deprived; 100 = most deprived).

Other clinical factors related to rejection are presented in Table 3. AMR was associated with younger recipient age, recipient pretransplant diabetes, repeat transplantation, pretransplant HLA-DSA, and recipient biological sex in univariable analyses; whereas donor age was significant only in multivariable models. TCMR was associated in both univariable and multivariable analyses with non–White European ethnicity, recipient pretransplant diabetes, transplant year, the number of HLA-ABDR mismatches, donor type (donation after brain death, donation after circulatory death, living), pretransplant HLA-DSA, and baseline immunosuppression; recipient age was significant only in multivariable models.

### Effect of Deprivation Domains on Posttransplant Outcomes

We independently analyzed the parameters comprising the BIMD (income, employment, crime, housing, education, and health) in relation to the outcomes, again using Cox as well as Fine and Gray models. For all-cause graft failure, a significant association was found with income (only univariable) (HR: 1.05, 95% CI: 1.01–1.09, *P* = 0.025), housing (only univariable) (HR: 1.06, 95% CI: 1.01–1.12, *P* = 0.030) and health (univariable and multivariable) (HR: 1.11, 95% CI: 1.02–1.19, *P* = 0.012 and aHR 1.11, 95% CI: 1.03–1.21, *P* = 0.009).

No significant associations were observed with graft failure (Supplementary Table S4). However, the mortality rate and its cumulative incidence were significantly associated with housing, in both univariable (HR: 1.09, 95% CI: 1.02–1.16, *P* = 0.015 and subdistribution HR: 1.08, 95% CI: 1.01–1.15, *P* = 0.023) and multivariable analyses (HR: 1.07, 95% CI: 1.00–1.15, *P* = 0.045 and subdistribution HR 1.08, 95% CI 1.01–1.15, *P* = 0.023). Rejection was significantly associated with employment (HR: 1.10, 95% CI: 1.04–1.15, *P* = 0.001) and education (HR: 1.07, 95% CI: 1.03–1.11, *P* = 0.001) in both univariable and multivariable models. With crime, only a univariable association was found (HR: 1.04, 95% CI: 1.00–1.08, *P* = 0.041) (Supplementary Table S5). Concerning rejection phenotypes, TCMR was independently associated with employment (HR: 1.11, 95% CI: 1.05–1.17, *P* < 0.001), crime (HR: 1.04, 95% CI: 1.01–1.09, *P* = 0.029), and education (HR: 1.06, 95% CI: 1.02–1.10, *P* = 0.003), whereas AMR showed no significant association with any of the domains.

## Discussion

This single-center observational cohort study demonstrated the impact of socioeconomic deprivation on kidney transplant outcomes. Using the BIMD, a robust area-based deprivation index, we found that socioeconomic deprivation was significantly associated with higher rates of rejection, and more specifically with TCMR. All-cause graft failure showed a significant effect in univariable analyses; however, this did not persist after multivariable adjustment, and no effects were observed for graft failure or mortality separately. Analysis of the individual BIMD domains (income, employment, crime, housing, education, and health) showed that housing was related to mortality, whereas employment, crime, and education were related to rejection (TCMR).

The relation between socioeconomic deprivation and kidney transplant outcomes has been explored in several international studies, with mixed results. In the US, where health care is primarily privatized, high socioeconomic deprivation was linked to poorer graft survival in pediatric transplants.^17^ In adult populations, lower education levels, reduced income, and lack of private insurance were found to be independent risk factors of shorter allograft and patient survival time.^6^^,^^18^, ^19^, ^20^ Stephens *et al.*^8^ investigating a UK cohort, reported higher rates of acute rejection and poorer graft survival with increasing overall deprivation. Our findings partially align, because we observed significantly higher rejection rates in more deprived patients; however, no significant impact on graft survival was identified. Similarly, Begaj *et al.*^7^ (UK) demonstrated that the least deprived recipients had a significantly reduced risk of death at 1 and 5 years posttransplant, a finding that could not be replicated in our study. In Ireland, where government-funded services are combined with a reliance on private insurance, Ward *et al.*^21^ reported no differences in patient or allograft survival across socioeconomic groups. Likewise, Aitken *et al.*^22^ (Scotland) and Laging *et al.*^23^ (The Netherlands), both representing countries with universal health care systems similar to Belgium, found no evidence of socioeconomic disparities affecting allograft or patient outcomes after kidney transplantation.

In the US, insurance coverage for immunosuppressive medications after kidney transplantation has long been limited. Medicare,^24^ the main public insurer for patients with end-stage kidney disease, historically covered these drugs for only 3 years posttransplant, unless the recipient already qualified through age or disability. Consequently, many younger patients lost coverage while their graft was still functioning, increasing the risk of nonadherence and graft loss. A 2000 policy^25^ change extending coverage to the life of the graft for Medicare-eligible recipients was associated with marked improvements in graft survival, highlighting the importance of sustained medication access. Since 2023, lifetime coverage has been available for patients without other insurance; however, eligibility remains restricted and far from universal. In Belgium, by contrast, health insurance is mandatory and provides lifelong coverage for immunosuppressive therapy.

The association we observed between socioeconomic deprivation and rejection was explained by an increased hazard of TCMR, whereas no significant association was detected with AMR. Our data do not allow us to assess the mechanisms behind the higher rejection risks among recipients from socioeconomically deprived areas. However, it could be hypothesized that noncompliance with immunosuppressive regimens is a primary factor in this disparity. In addition, psychosocial factors associated with deprivation, such as increased stress, reduced social support, limited access to health care resources, decreased health literacy, and financial constraints may indirectly affect health through medically negative behaviors such as smoking, excessive alcohol consumption, and unhealthy eating.^8^^,^^26^ Although challenging to prove, these factors provide a plausible explanation for the observed disparities in overall rejection and TCMR rates. The unexplained lack of association between AMR and socioeconomic deprivation is noteworthy. Importantly, pretransplant DSA, a key risk factor for AMR, did not differ across deprivation groups, reflecting its consistent avoidance in the allocation process for all patients. Moreover, the relatively low incidence of AMR in our cohort (13.7% at 5 years) may have provided limited statistical power to detect a significant association. Notwithstanding, this is the first study to investigate the link between socioeconomic deprivation and rejection subtypes.

HRs for all-cause graft failure, graft failure, and mortality were of similar magnitude, in the range of 1.05 to 1.07, whereas these for rejection were approximately 1.10 to 1.11. All-cause graft failure was significantly related to deprivation in univariable but not in multivariable analyses and we could not confirm this association for graft failure or mortality separately, indicating that the composite end point likely reflects greater power from more events. At the same time, the significant approximately 10% hazard increase for rejection is almost double of the (nonsignificant) approximately 5% increase for graft failure or mortality, suggesting that this difference is not only attributable to sample size. Because TCMR in our cohort occurred mainly early after transplantation, its long-term impact on graft failure or mortality may be limited, which emphasizes the difference between these outcomes.

Our study found that the most deprived recipients were more likely to be of other than White European ethnicity, consistent with previous research indicating that non-White individuals are disproportionately represented in areas of high socioeconomic deprivation.^7^ Three US studies identified race and ethnicity as independent predictors of graft survival among kidney transplant recipients,^27^, ^28^, ^29^ though we did not observe this association in our cohort, likely because of a lack of power. However, we observed an increased occurrence of TCMR in recipients of other than White European ethnicity, even after adjusting for BIMD scores, suggesting that ethnicity and socioeconomic deprivation both exert an independent influence on the occurrence of rejection. This highlights the complex interplay of biological, immunological, and societal factors in transplant outcomes. Possible explanations include differences in immune response, immunosuppressive metabolism, and access to care. This underscores the need for further investigation into ethnic disparities in transplantation and targeted strategies to mitigate modifiable risk factors, particularly among socioeconomically disadvantaged populations.

We identified associations between mortality and housing, as well as between rejection and education, employment, and crime. Housing is defined by metrics including the proportion of tenants in the statistical sector and the availability of basic amenities, such as heating and a bathroom.^13^ This domain showed a weak correlation with the overall BIMD score. The observed association between housing and mortality may therefore reflect the broader impact of living conditions on long-term health outcomes. Both education (defined as the proportion of early school leavers) and employment (defined as the proportion of unemployed individuals)^13^ were strongly correlated with the BIMD score, suggesting that they largely contribute to the observed correlation between BIMD and rejection. Both parameters have been linked to graft and patient survival in previous studies^6^^,^^30^; however, we were unable to confirm this association, making our identified association with rejection not straightforward. The link between rejection and education or employment likely reflects a combination of behavioral, psychosocial, and health care access–related factors. Lower health literacy and unemployment may lead to inconsistent medication adherence because of financial constraints or disruptions in daily routines affecting medication schedules. Psychosocial stress can increase nonadherence and promote high-risk behavior. Despite universal health care, lower education and unemployment may result in fewer follow-ups, delayed symptom recognition, and reduced health care engagement, thereby increasing the risk of undiagnosed early rejection signs. Employment status influences social support, with unstable jobs or workplace stress further limiting medical appointment attendance and posttransplant care. Moreover, interpreting employment data in the transplantation context is complex, because transplantation often influences employment status. For instance, Helantera *et al.*^31^ reported increased employment opportunities after kidney transplantation compared with in-center hemodialysis, which may enhance income and overall well-being. Although the direct connection between crime and rejection is unclear, it may indirectly reflect socioeconomic disadvantages such as unemployment, which is known to negatively impact health outcomes.

This analysis has several limitations. First, the number of graft failure and death with functioning graft events was lower than for rejection, which could have reduced statistical power and precision for these outcomes, potentially obscuring modest associations with deprivation. Second, the BIMD evaluates deprivation at the statistical sector level rather than at the individual level, which may introduce ecological bias. Not all residents of a deprived area are necessarily disadvantaged. Furthermore, the dynamic nature of residence adds complexity, because individuals may move into or out of a specific area. However, a key strength of this study is that the deprivation index used encompasses multiple factors, providing a very comprehensive assessment of deprivation.^32^ Second, several confounders likely impact posttransplantation outcomes but could not be assessed. These include psychosocial factors (such as adherence, mental health, and support networks), unmeasured donor and recipient health conditions (e.g., donor diabetes), dialysis duration, and cause of renal failure, all of which may affect rejection risk and other outcomes. Third, this study did not address access to transplantation, which has been shown to vary by socioeconomic status.^33^, ^34^, ^35^, ^36^ Lastly, our results may not be generalizable to countries with different health care systems, where socioeconomic deprivation could have a weaker or stronger influence on outcomes.

In conclusion, in Belgium, socioeconomic deprivation, as measured using the comprehensive BIMD, was associated with higher rejection rates, particularly TCMR. Although an association with all-cause graft failure was observed in univariable analyses, this did not persist after multivariable adjustment, and no independent associations were observed with graft failure or mortality in cause-specific and competing risks analyses. These findings indicate that universal health care may reduce, but not fully neutralize, the impact of socioeconomic deprivation on posttransplant outcomes. Addressing the complex social, behavioral, and health care access factors underlying these disparities, and determining whether deprivation limits access to transplantation should be priorities for future research and policy.

## Disclosure

All the authors declared no competing interests.

## Supplementary Material

Supplementary File (PDF) **Figure S1.** Flowchart study population. **Figure S2.** Distribution of BIMD score and domains across deprivation groups. **Figure S3.** Linear regression of the BIMD domains on the overall BIMD score. **Figure S4.** Schoenfeld residual plot to assess non-proportionality of the BIMD score effect on (A) graft failure, (B) death with functioning graft, (C) all-cause graft failure and (D) overall rejection. **Figure S5.** Martingale residual plot to assess non-linearity of the BIMD score effect on (A) graft failure, (B) death with functioning graft, (C) all-cause graft failure and (D) overall rejection. **Table S1.** BIMD Domains and their indicators. **Table S2.** STROBE guidelines. **Table S3.** Association of BIMD score with all-cause graft failure (*n* = 1870). **Table S4.** Association of BIMD domains with all-cause graft failure, graft failure and mortality. **Table S5.** Association of BIMD domains with overall rejection, TCMR and AMR.
