# Supplementary material for: Socioeconomic Deprivation and Kidney Transplant Outcomes
Source: Kidney Int Rep. 2025 Nov 5;11(2):103667. doi: 10.1016/j.ekir.2025.10.024 (PMC12769133; doi:10.1016/j.ekir.2025.10.024)
Supplement: Supplementary File (PDF) — Figure S1. Flowchart study population. Figure S2. Distribution of BIMD score and domains across deprivation groups. Figure S3. Linear regression of the BIMD domains on the overall BIMD score. Figure S4. Schoenfeld residual plot to assess non-proportionality of the BIMD score effect on (A) graft failure, (B) death with functioning graft, (C) all-cause graft failure and (D) overall rejection. Figure S5. Martingale residual plot to assess non-linearity of the BIMD score effect on (A) graft failure, (B) death with functioning graft, (C) all-cause graft failure and (D) overall rejection. Table S1. BIMD Domains and their indicators. Table S2. STROBE guidelines. Table S3. Association of BIMD score with all-cause graft failure (n = 1870). Table S4. Association of BIMD domains with all-cause graft failure, graft failure and mortality. Table S5. Association of BIMD domains with overall rejection, TCMR and AMR. [file mmc1.pdf]

Supplementary Material

Table S1. BIMD Domains and their indicators.
This table provides an overview of the BIMD domains and the specific indicators that define each domain, adapted from Otavova et al.[13]

Domains and their Indicators
**Education (25%)**
• NEET indicator[a]<br>• Proportion of<br>○ working age adults without qualification<br>○ early school leavers
**Employment (20%)**
• Proportion of working age population who is<br>○ unemployed<br>○ not working due to disability
**Income (20%)**
• Proportion of<br>○ individuals in the lowest income decile<br>○ households with net taxable income below the minimum living wage
**Housing (15%)**
• Proportion of tenants<br>• Proportion of individuals living in dwellings<br>○ less than 0.5 room/person<br>○ without central heating<br>○ without bathroom
**Health (15%)**
• Preventable mortality fraction<br>• Standardized<br>○ mortality ratio<br>○ suicide rate
**Crime (5%)**
• Rate of<br>○ property crimes<br>○ violent crimes<br>○ family violence crimes<br>○ fraud

[a] Young people Neither in Employment nor in Education or Training[35]

Table S2. STROBE guidelines.

	Item No	Recommendation	Page No
**Title and abstract**	1	(*a*) Indicate the study's design with a commonly used term in the title or the abstract	1
		(*b*) Provide in the abstract an informative and balanced summary of what was done and what was found	2
**Introduction**			
Background/rationale	2	Explain the scientific background and rationale for the investigation being reported	3
Objectives	3	State specific objectives, including any prespecified hypotheses	3
**Methods**			
Study design	4	Present key elements of study design early in the paper	4
Setting	5	Describe the setting, locations, and relevant dates, including periods of recruitment, exposure, follow-up, and data collection	4–5
Participants	6	(*a*) Give the eligibility criteria, and the sources and methods of selection of participants. Describe methods of follow-up	4–5
Variables	7	Clearly define all outcomes, exposures, predictors, potential confounders, and effect modifiers. Give diagnostic criteria, if applicable	4–5
Data sources/ measurement	8*	For each variable of interest, give sources of data and details of methods of assessment (measurement). Describe comparability of assessment methods if there is more than one group	4
Bias	9	Describe any efforts to address potential sources of bias	5–6
Study size	10	Explain how the study size was arrived at	4–5
Quantitative variables	11	Explain how quantitative variables were handled in the analyses. If applicable, describe which groupings were chosen and why	5–6
Statistical methods	12	(*a*) Describe all statistical methods, including those used to control for confounding	5–6
		(*b*) Describe any methods used to examine subgroups and interactions	5–6
		(*c*) Explain how missing data were addressed	5–6
		(*d*) If applicable, explain how loss to follow-up was addressed	5–6
		(*e*) Describe any sensitivity analyses	5–6
**Results**			
Participants	13*	(a) Report numbers of individuals at each stage of study—eg numbers potentially eligible, examined for eligibility, confirmed eligible, included in the study, completing follow-up, and analysed	7
		(b) Give reasons for non-participation at each stage	7
		(c) Consider use of a flow diagram	7, Figure S1
Descriptive data	14*	(a) Give characteristics of study participants (eg demographic, clinical, social) and information on exposures and potential confounders	7
		(b) Indicate number of participants with missing data for each variable of interest	7
		(c) Summarise follow-up time (eg, average and total amount)	7
Outcome data	15*	Report numbers of outcome events or summary measures over time	7–9
Main results	16	(*a*) Give unadjusted estimates and, if applicable, confounder-adjusted estimates and their precision (eg, 95% confidence	7–9

		interval). Make clear which confounders were adjusted for and why they were included	
		(*b*) Report category boundaries when continuous variables were categorized	5, Figure 1
		(*c*) If relevant, consider translating estimates of relative risk into absolute risk for a meaningful time period	7–9, Figure 2
Other analyses	17	Report other analyses done—eg analyses of subgroups and interactions, and sensitivity analyses	8–10, Table S3–S4
**Discussion**			
Key results	18	Summarise key results with reference to study objectives	11
Limitations	19	Discuss limitations of the study, taking into account sources of potential bias or imprecision. Discuss both direction and magnitude of any potential bias	14
Interpretation	20	Give a cautious overall interpretation of results considering objectives, limitations, multiplicity of analyses, results from similar studies, and other relevant evidence	11–14
Generalisability	21	Discuss the generalisability (external validity) of the study results	14
**Other information**			
Funding	22	Give the source of funding and the role of the funders for the present study and, if applicable, for the original study on which the present article is based	15

Table S3. Association of BIMD score with all-cause graft failure (n=1870).
Hazard ratios for all-cause graft failure, up to 10 years post-transplantation, including the BIMD score as a continuous variable (0 = least deprived; 100 = most deprived).

	Cox model			
**All-cause graft failure**	**Univariable HR (95%CI)**	**p-value**	**Multivariable HR (95%CI)**	**p-value**
BIMD score, per 10	1.07 (1.00–1.14)	0.043	1.03 (0.97–1.10)	0.315
Recipient age per 10 years	1.53 (1.42–1.65)	<0.001	1.40 (1.29–1.52)	<0.001
Recipient female	0.99 (0.84–1.16)	0.879	0.96 (0.81–1.14)	0.645
Other ethnicity (ref= White European)	1.03 (0.63–1.69)	0.912	0.98 (0.58–1.64)	0.939
Recipient re-transplant diabetes	2.16 (1.81–2.58)	<0.001	1.79 (1.49–2.15)	<0.001
Repeat transplantation	1.42 (1.16–1.74)	0.001	1.67 (1.33–2.10)	<0.001
Transplant year	1.02 (1.00–1.04)	0.137	1.00 (0.98–1.03)	0.728
Donor age, per 10 years	1.22 (1.15–1.29)	<0.001	1.08 (1.02–1.15)	0.015
Donor female	0.94 (0.80–1.10)	0.442	1.04 (0.88–1.22)	0.659
Living donation (ref=DBD)	0.36 (0.23–0.58)	<0.001	0.52 (0.32–0.84)	0.007
Donor DCD (ref= DBD)	0.79 (0.64–0.99)	0.037	0.83 (0.67–1.04)	0.109
Pre-transplant HLA-DSA	1.77 (1.39–2.25)	<0.001	1.47 (1.13–1.91)	0.004
No. of HLA-ABDR mm	1.16 (1.08–1.23)	<0.001	1.10 (1.03–1.17)	0.007
TAC-MMF-CS	1.03 (0.63–1.69)	0.912	1.18 (0.87–1.61)	0.280

Abbreviations: AMR, antibody-mediated rejection; BIMD, Belgian index of multiple deprivation; CS, corticosteroids; DBD, donation after brain death; DCD, donation after circulatory death; HLA-DSA, anti-human leukocyte antigen donor-specific antibodies; IQR, interquartile range; MMF, mycophenolate mofetil; SD, standard deviation; TAC, tacrolimus.

Table S4. Association of BIMD domains with all-cause graft failure, graft failure and mortality.
Hazard ratios for all-cause graft failure, and hazard and subdistribution hazard ratios for graft failure and mortality up to 10 years post-transplantation, are shown for the different BIMD domains (n=1870). For each domain, a score of 0 represents the least deprivation and 100 the greatest deprivation.

	**Cox model**				**Fine and Gray**			
**All-cause graft failure**	**Univariable HR (95%CI)**	**p-value**	**Multivariable[a] HR (95%CI)**	**p-value**				
Income	1.05 (1.01–1.09)	0.025	1.02 (0.98–1.06)	0.277				
Employment	1.03 (0.97–1.08)	0.327	1.01 (0.96–1.06)	0.693				
Crime	1.02 (0.98–1.05)	0.398	1.01 (0.97–1.04)	0.777				
Housing	1.06 (1.01–1.12)	0.030	1.05 (0.99–1.11)	0.078				
Education	1.02 (0.98–1.05)	0.438	1.00 (0.96–1.04)	0.938				
Health	1.11 (1.02–1.19)	0.012	1.11 (1.03–1.21)	0.009				
**Graft failure**					**Univariable HR (95%CI)**	**p-value**	**Multivariable[a] HR (95%CI)**	**p-value**
Income	1.06 (1.00–1.12)	0.067	1.05 (0.98–1.11)	0.160	1.05 (0.99–1.12)	0.077	1.05 (0.99–1.11)	0.142
Employment	1.04 (0.96–1.13)	0.346	1.02 (0.94– 1.11)	0.652	1.04 (0.96–1.13)	0.352	1.02 (0.94–1.11)	0.621
Crime	1.01 (0.96–1.07)	0.628	1.01 (0.95–1.07)	0.760	1.01 (0.96–1.07)	0.680	1.01 (0.95–1.06)	0.834
Housing	1.02 (0.94–1.11)	0.611	1.01 (0.93–1.11)	0.759	1.01 (0.93–1.10)	0.795	1.00 (0.92–1.09)	0.981
Education	1.01 (0.95–1.07)	0.742	0.99 (0.94–1.05)	0.817	1.01 (0.95–1.07)	0.746	0.99 (0.94–1.05)	0.817
Health	1.11 (0.99–1.26)	0.075	1.12 (0.99–1.27)	0.068	1.10 (0.98–1.23)	0.103	1.11 (0.99–1.24)	0.088
**Mortality**								

Income	1.04 (0.98–1.09)	0.164	1.01 (0.96–1.07)	0.701	1.03 (0.98–1.08)	0.285	1.00 (0.95–1.05)	0.919
Employment	1.02 (0.95–1.09)	0.626	1.01 (0.95–1.09)	0.677	1.01 (0.95–1.08)	0.715	1.01 (0.94–1.07)	0.874
Crime	1.02 (0.97–1.06)	0.486	1.01 (0.96–1.06)	0.692	1.02 (0.97–1.06)	0.498	1.01 (0.96–1.06)	0.717
Housing	1.09 (1.02–1.16)	0.015	1.07 (1.00-1.15)	0.045	1.08 (1.01–1.15)	0.023	1.08 (1.01–1.15)	0.023
Education	1.02 (0.97–1.07)	0.461	1.01 (0.96–1.05)	0.797	1.02 (0.97–1.07)	0.460	1.01 (0.96–1.05)	0.758
Health	1.10 (0.99–1.22)	0.073	1.11 (0.99–1.24)	0.065	1.08 (0.98–1.19)	0.133	1.09 (0.98–1.21)	0.121

[a] Corrected for recipient age, recipient sex, ethnicity, recipient pre-transplant diabetes, repeated transplantations, transplant year, donor age, donor sex, donor type (DBD; DCD; living), pre-transplant DSA, HLA-ABDR mismatches and baseline immunosuppression.

Table S5. Association of BIMD domains with overall rejection, TCMR and AMR.

Hazard ratios for overall rejection, TCMR, and AMR up to 5 years post-transplantation, are shown for the different BIMD domains (n=1777). For each BIMD domain, the score 0 corresponds to the least deprivation, while 100 corresponds to the most deprivation.

	**Cox model**			
**Overall Rejection**	**Univariable HR (95%CI)**	**p-value**	**Multivariable[a] HR (95%CI)**	**p-value**
Income	1.04 (1.00–1.09)	0.061	1.02 (0.98–1.07)	0.386
Employment	1.10 (1.04–1.15)	<u>0.001</u>	1.07 (1.02–1.13)	<u>0.011</u>
Crime	1.04 (1.00–1.08)	<u>0.041</u>	1.03 (0.99–1.07)	0.095
Housing	0.98 (0.92–1.04)	0.504	0.98 (0.92–1.05)	0.612
Education	1.07 (1.03–1.11)	<u>0.001</u>	1.05 (1.01–1.09)	<u>0.008</u>
Health	0.94 (0.85–1.04)	0.209	0.95 (0.86–1.05)	0.349
**TCMR**				
Income	1.02 (0.97–1.07)	0.393	1.01 (0.96–1.06)	0.724
Employment	1.11 (1.05–1.17)	<u><0.001</u>	1.09 (1.03–1.15)	<u>0.003</u>
Crime	1.04 (1.01–1.09)	<u>0.029</u>	1.04 (1.00–1.08)	<u>0.038</u>
Housing	1.00 (0.93–1.06)	0.901	1.00 (0.94–1.07)	0.944
Education	1.06 (1.02–1.10)	<u>0.003</u>	1.05 (1.01–1.09)	<u>0.016</u>
Health	0.96 (0.87–1.06)	0.428	0.98 (0.88–1.08)	0.676
**AMR**				
Income	1.05 (0.97–1.13)	0.203	0.99 (0.91–1.08)	0.849
Employment	1.07 (0.97–1.17)	0.192	1.02 (0.93–1.13)	0.652
Crime	1.00 (0.94–1.07)	0.918	1.01 (0.94–1.08)	0.847
Housing	0.98 (0.88–1.10)	0.769	0.95 (0.84–1.07)	0.377
Education	1.04 (0.97–1.12)	0.233	1.02 (0.94–1.09)	0.670
Health	0.89 (0.74–1.07)	0.216	0.92 (0.75–1.12)	0.392

[a] Corrected for recipient age, recipient sex, ethnicity, recipient pre-transplant diabetes, repeated transplantations, transplant year, donor age, donor sex, donor type (DBD; DCD; living), pre-transplant DSA, HLA-ABDR mismatches and baseline immunosuppression.

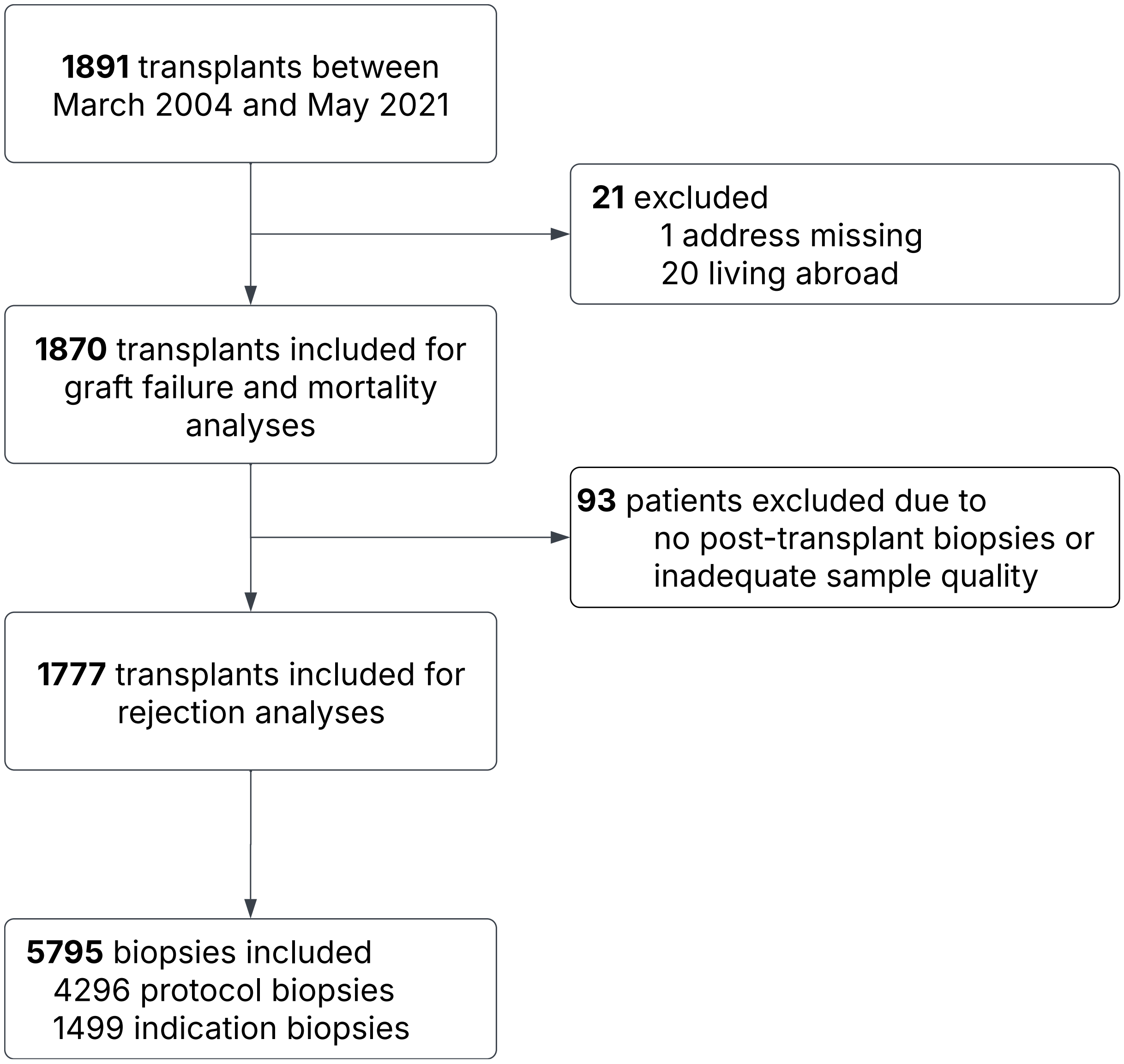

Figure S1. Flowchart Study Population.

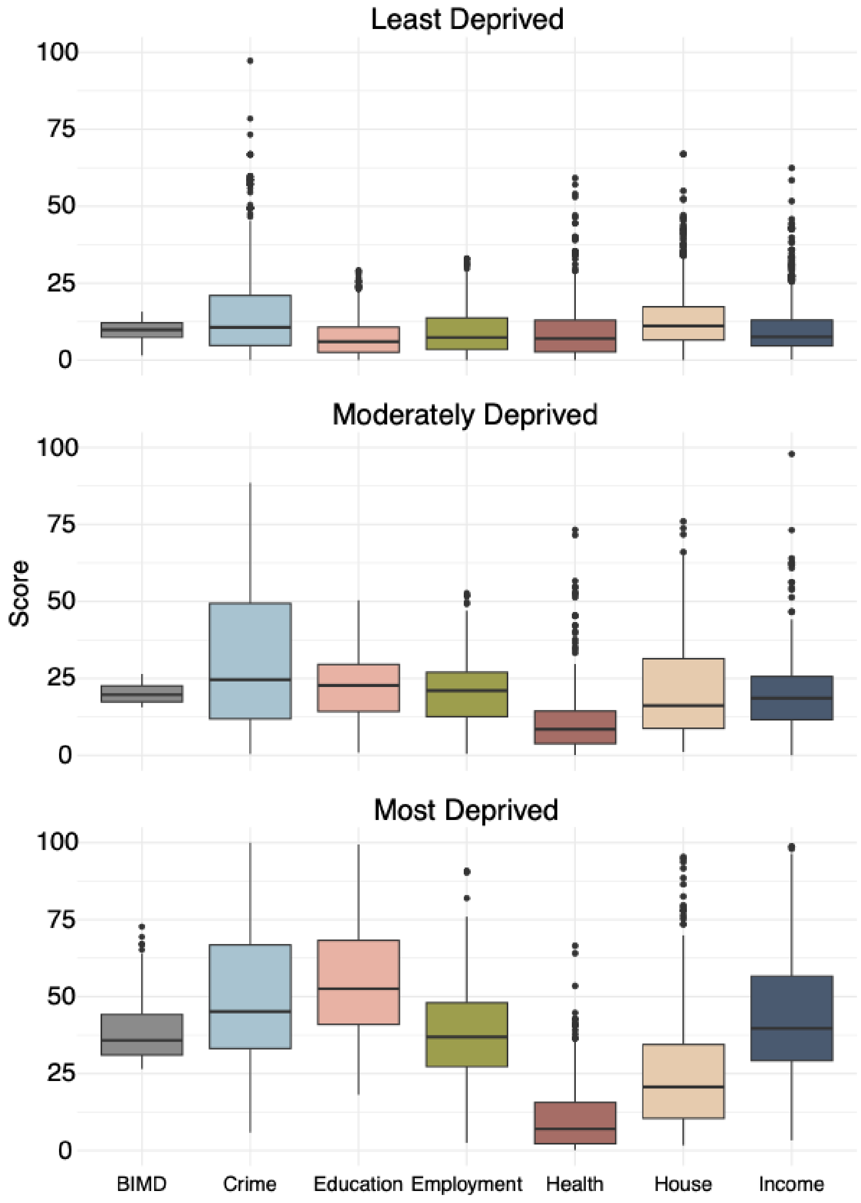

Figure S2. Distribution of BIMD score and domains across deprivation groups.
Boxplot showing the distribution of the total BIMD score and its domains across the three deprivation groups: Least deprived (n=1060), Moderately deprived (n=463), and Most deprived (n=347).

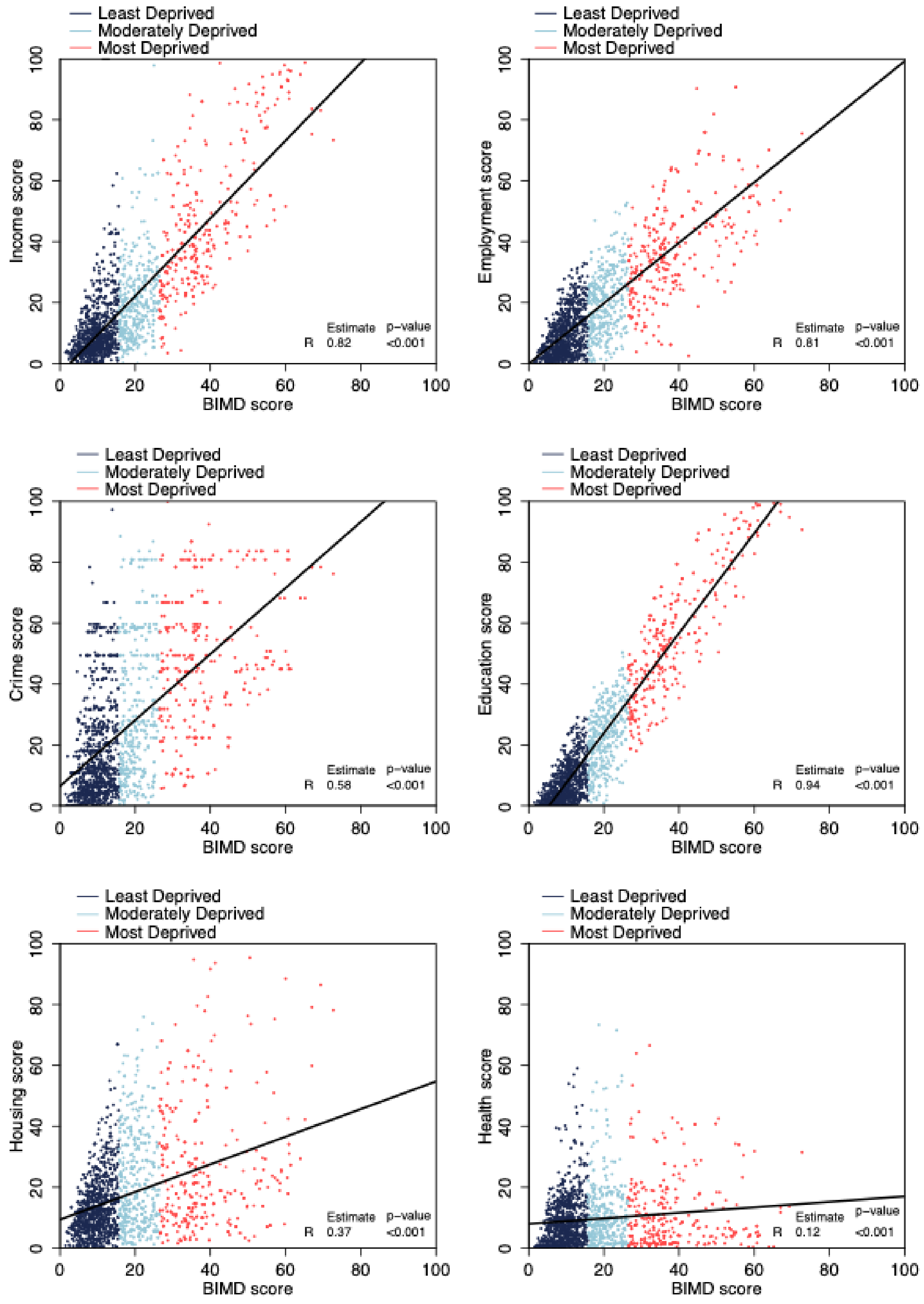

Figure S3. Linear regression of the BIMD domains on the overall BIMD score.
R represents the Pearson correlation. A BIMD score of 100 corresponds to the most deprived, while 0 corresponds the least deprived. Similarly, for each BIMD domain, the score 0 corresponds to the least deprivation, while 100 corresponds to the most deprivation.

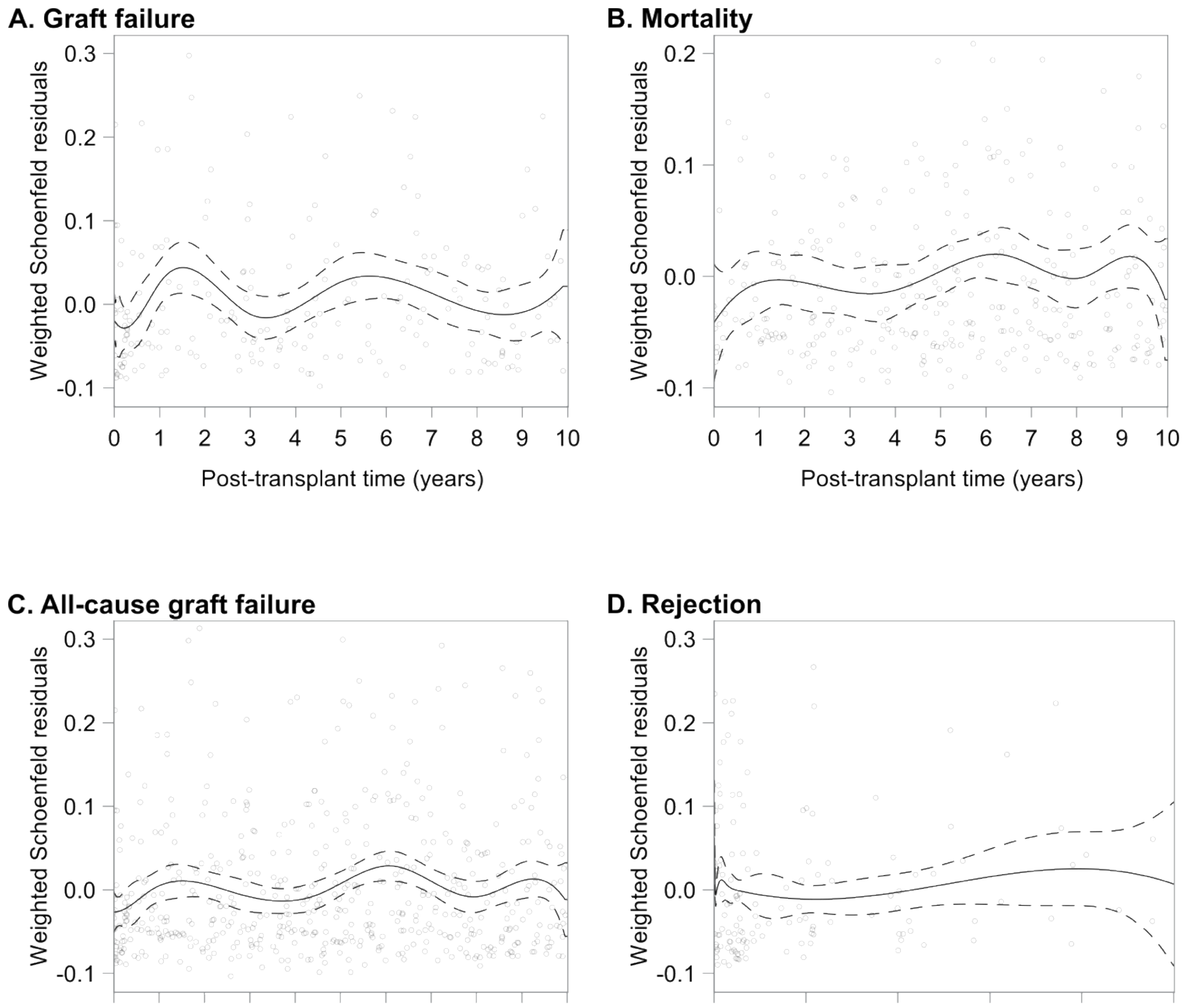

Figure S4. Schoenfeld residual plot to assess non-proportionality of the BIMD score effect on (A) graft failure, (B) death with functioning graft, (C) all-cause graft failure and (D) overall rejection. Deviations from a horizontal line were visually assessed via including a B-spline function of degree 3 with 5 knots.

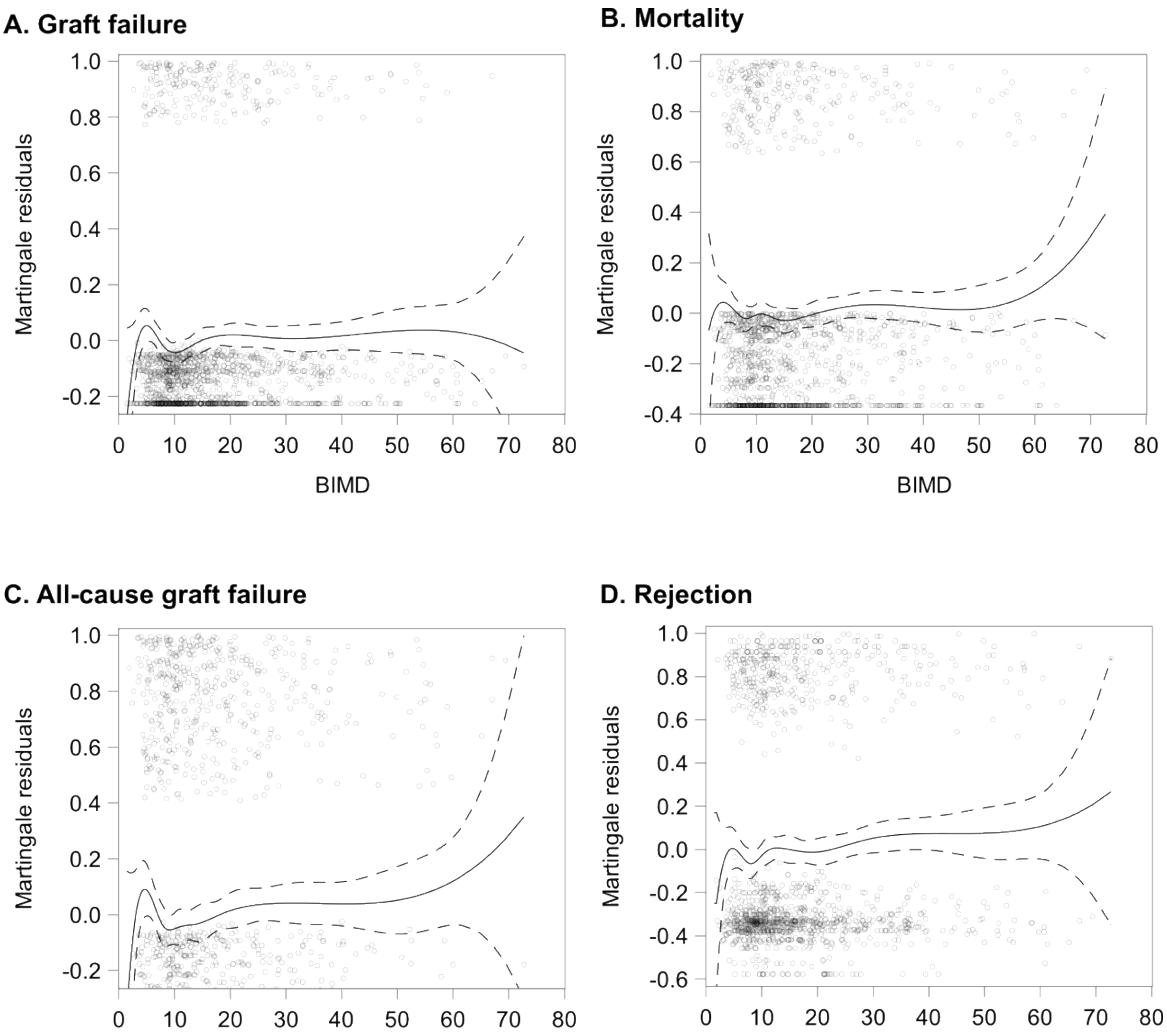

Figure S5. Martingale residual plot to assess non-linearity of the BIMD score effect on (A) graft failure, (B) death with functioning graft, (C) all-cause graft failure and (D) overall rejection. Deviations from a horizontal line were visually assessed via including a B-spline function of degree 3 with 5 knots.
